# *Molecules* Best Paper Award 2015

**DOI:** 10.3390/molecules20011751

**Published:** 2015-01-20

**Authors:** Derek J. McPhee

**Affiliations:** Editor-in-Chief, MDPI AG, Klybeckstrasse 64, Basel CH-4057, Switzerland; E-Mail: mcphee@mdpi.com

*Molecules* instituted some years ago a “Best Paper” award to recognize the most outstanding papers in the area of organic synthesis, natural products, medicinal chemistry and molecular diversity published each year in *Molecules*. We are pleased to announce the third “*Molecules* Best Paper Award” for 2015. The winners were chosen by the Editor-in-Chief and selected editorial board members from among all the papers published in 2011. Reviews and research papers were evaluated separately. We are pleased to announce that the following eight papers have won the *Molecules* Best Paper Award for 2015:

## Article Award:

### 1st Prize

**Ulrike Endesfelder, Sebastian Malkusch, Benjamin Flottmann, Justine Mondry, Piotr Liguzinski, Peter J. Verveer and Mike Heilemann**

Chemically Induced Photoswitching of Fluorescent Probes—A General Concept for Super-Resolution Microscopy

*Molecules* **2011**, *16*(4), 3106-3118; doi:10.3390/molecules16043106

Available online: http://www.mdpi.com/1420-3049/16/4/3106

### 2nd Prize

**Victor Alves Carneiro, Hélcio Silva dos Santos, Francisco Vassiliepe Sousa Arruda, Paulo Nogueira Bandeira, Maria Rose Jane Ribeiro Albuquerque, Maria Olívia Pereira, Mariana Henriques, Benildo Sousa Cavada and Edson Holanda Teixeira**

Casbane Diterpene as a Promising Natural Antimicrobial Agent against Biofilm-Associated Infections

*Molecules* **2011**, *16*(1), 190-201; doi:10.3390/molecules16010190

Available online: http://www.mdpi.com/1420-3049/16/1/190

### 3nd Prize

**Yasunori Yamamoto, Tomohiko Shirai, Momoko Watanabe, Kazunori Kurihara and Norio Miyaura**

Ru/Me-BIPAM-Catalyzed Asymmetric Addition of Arylboronic Acids to Aliphatic Aldehydes and alpha-Ketoesters

*Molecules* **2011**, *16*(6), 5020-5034; doi:10.3390/molecules16065020

Available online: http://www.mdpi.com/1420-3049/16/6/5020

**Sekelwa Cosa, Leonard V. Mabinya, Ademola O. Olaniran, Omobola O. Okoh, Kim Bernard, Shaun Deyzel and Anthony I. Okoh**

Bioflocculant Production by *Virgibacillus sp.* Rob Isolated from the Bottom Sediment of Algoa Bay in the Eastern Cape, South Africa

*Molecules* **2011**, *16*(3), 2431-2442; doi:10.3390/molecules16032431

Available online: http://www.mdpi.com/1420-3049/16/3/2431

**Abdelaaty A. Shahat, Abeer Y. Ibrahim, Saber F. Hendawy, Elsayed A. Omer, Faiza M. Hammouda, Fawzia H. Abdel-Rahman and Mahmoud A. Saleh**

Chemical Composition, Antimicrobial and Antioxidant Activities of Essential Oils from Organically Cultivated Fennel Cultivars

*Molecules* **2011**, *16*(2), 1366-1377; doi:10.3390/molecules16021366

Available online: http://www.mdpi.com/1420-3049/16/2/1366

## Review Award:

### 1st Prize

**Chad C. Eichman and James P. Stambuli**

Transition Metal Catalyzed Synthesis of Aryl Sulfides

*Molecules* **2011**, *16*(1), 590-608; doi:10.3390/molecules16010590

Available online: http://www.mdpi.com/1420-3049/16/1/590

### 2nd Prize

**Michael Oelgemöller and Oksana Shvydkiv**

Recent Advances in Microflow Photochemistry

*Molecules* **2011**, *16*(9), 7522-7550; doi:10.3390/molecules16097522

Available online: http://www.mdpi.com/1420-3049/16/9/7522

### 3nd Prize

**Anna K. Jäger and Lasse Saaby**

Flavonoids and the CNS

*Molecules* **2011**, *16*(2), 1471-1485; doi:10.3390/molecules16021471

Available online: http://www.mdpi.com/1420-3049/16/2/1471

The prize awarding committee recognized the article “Chemically Induced Photoswitching of Fluorescent Probes—A General Concept for Super-Resolution Microscopy” as “….a good addition to the science of super resolution imaging both in fixed and in live cells” since it “….describes the fluorescence properties of five fluorescent proteins together with organic fluorophores and the reversible photoswitching properties of mEos2 was studied in more detail.” The review “Transition Metal Catalyzed Synthesis of Aryl Sulfides” provided “…a useful adjunct to the library of C-S bond syntheses”, as “…it describes transition metal catalyzed synthesis of aryl sulfides” and “… some of aryl sulfides occur in biologically active compounds.”

We believe these eight exceptional papers represent valuable contributions to *Molecules* and the scientific literature. On behalf of the Prize Awarding Committee and the Editorial Board of *Molecules*, we would like to congratulate these eight teams for their excellent work. In recognition for their accomplishment, Dr. Mike Heilemann, Dr. Victor Alves Carneiro, Dr. Yasunori Yamamoto, Dr. Anthony I. Okoh and Dr. Mahmoud A. Saleh will receive prizes of 1,000, 800, 600, 400 and 200 CHF, respectively, and the privilege of publishing an additional Open Access format paper of their choice free of charge in *Molecules*. Dr. James P. Stambuli, Dr. Michael Oelgemöller and Dr. Anna K. Jäger will be awarded the privilege of publishing an additional research paper free of charge in Open Access format in *Molecules*.

## *Prize* *Awarding* *Committee* 

### Editor-in-Chief

**Dr. Derek J. McPhee **

MDPI AG, Klybeckstrasse 64, Basel CH-4057, Switzerland

E-Mail: mcphee@mdpi.com

### Associate Editor

**Prof. Dr. Fernando Albericio**

^1^ School of Chemistry, Yachay Tech, Yachay City of Knowledge, 100119-Urcuqui, Ecuador

^2^ Institute for Research in Biomedicine (IRB Barcelona), Parc Científic de Barcelona, Baldiri Reixac 10, 08028 Barcelona, Spain

^3^ School of Chemistry, University of KwaZulu-Natal, Durban 4001, South Africa

E-Mail: albericio@irbbarcelona.org

### Associate Editor

**Dr. Jean Jacques Vanden Eynde**

Laboratory of Organic Chemistry (FS), University of Mons-UMONS, Place du parc, 23, 7000 Mons, Belgium; E-Mail: jean-jacques.vandeneynde@ex.umons.ac.be

### Associate Editor

**Dr. John A. Beutler**

Molecular Targets Laboratory, Bldg 1052 Rm 110 NCI at Frederick, Frederick, MD 21702-1201, USA

E-Mail: beutlerj@mail.nih.gov

### Associate Editor

**Prof. Dr. Maurizio Battino**

Department of Odontostomatologic and Specialized Clinical Sciences, Sez-Biochimica, Faculty of Medicine, Università Politecnica delle Marche, Via Ranieri 65, 60100 Ancona, Italy

E-Mail: m.a.battino@univpm.it

### Editorial Board Member*, Section “Medicinal Chemistry”*

**Prof. Dr. Jarkko Rautio**

School of Pharmacy, University of Eastern Finland, P.O. Box 1627, 70211 Kuopio, Finland

E-Mail: jarkko.t.rautio@uef.fi

### Editorial Board Member*, Section “Organic Synthesis”*

**Prof. Dr. Osmo E. O. Hormi**

Department of Chemistry, University of Oulu, Linnanmaa, P.O.Box 333, FIN-90570 Oulu, Finland

E-Mail: osmo.hormi@oulu.fi

